# Hypertension Impact on Health-Related Quality of Life: A Cross-Sectional Survey among Middle-Aged Adults in Chongqing, China

**DOI:** 10.1155/2016/7404957

**Published:** 2016-08-17

**Authors:** Xianglong Xu, Yunshuang Rao, Zumin Shi, Lingli Liu, Cheng Chen, Yong Zhao

**Affiliations:** ^1^School of Public Health and Management, Chongqing Medical University, Chongqing 400016, China; ^2^Research Center for Medicine and Social Development, Chongqing Medical University, Chongqing 400016, China; ^3^The Innovation Center for Social Risk Governance in Health, Chongqing Medical University, Chongqing 400016, China; ^4^School of Nursing, Chongqing Medical University, Chongqing 400016, China; ^5^School of Medicine, Faculty of Health Sciences, The University of Adelaide, North Terrace, Adelaide, SA 5005, Australia

## Abstract

Hypertension is a major risk factor of cardiovascular disease in China, and yet little is known about health-related quality of life (HRQOL) and its associations with demographic and social-economic characteristics in middle-aged patients with hypertension. A cross-sectional survey was undertaken in Chongqing, China, using a multistage stratified random sampling methodology. Data was collected on 1,224 eligible adults, aged between 45 and 53 years, including the Medical Outcomes Survey Short Form-36 to measure HRQOL. Hypertension was associated with poor state of physical functioning, role-physical, bodily pain, general health, vitality, and social function (*p* < 0.05 for all). In multivariable analyses, education level, job conditions, average monthly income, smoking status, sleep quality, perception of relationship with family, childhood breastfeeding history, and body mass index were associated with domains of SF36 among those with hypertension (*p* < 0.05 for all). Hypertensive respondents with high education, marital status, breastfeeding, higher incomes, good quality of sleep, positive relationship with family, and higher body mass index have better HRQOL in middle-aged people with hypertension. Those unemployed had a better state of general health and had a poorer state of social function. Nonsmokers had a poorer state of bodily pain than smokers. This study provides detailed information of the implications for health care providers to gain a more complete picture of their hypertension patients' health.

## 1. Introduction

Hypertension is a well-known independent risk factor for many chronic diseases including diabetes and cardiovascular diseases causing a significant burden to the society and families [1]. Hypertension has been identified as the second leading risk factor in China, which accounted for 12.0% of disability-adjusted life years and 24.6% of deaths in 2010 [2]. According to the 2015 Chinese Chronic Disease and Nutrition Report, the prevalence of hypertension among adults aged 45–59 years and over 60 was 35.7% and 58.9% in 2012, respectively [3]. The incidence of hypertension has increased from 2.9 per 100 person-years in 1991–1997 to 5.3 per 100 person-years in 2004–2009 [4]. A national survey conducted in 2011-2012 found that nearly 40% of Chinese people aged 45 years or older were hypertensive [5]. However, the prevalence of awareness, treatment, and control was low: 44.2%, 38.0%, and 13.1% among those aged 45–59 years, respectively and 53.7%, 48.8%, and 16.1%, among those aged 60 years and above, respectively [3].

Health-related quality of life (HRQOL) has become increasingly important in clinical research over the last 15 years [6]. It provides a multidimensional perspective encompassing a patient's emotional, physical, and social functioning [7]. HRQOL is related to an individual's perception of the position in life in the context of culture and value systems and is influenced in a complex way by the person's physical health, psychological state, level of independence, and social relationships [8]. Hypertension is closely related to psychological and emotional problems, particularly in severe life stresses [9]. Studies have found that people with hypertension had a poorer quality of life indicator than people without the condition [10–13]. By establishing a proven link between the disease and HRQOL, then developing interventions programs aiming at improving HRQOL will become a new relevant therapeutic objective in hypertensive subjects [11].

Middle age is a special stage of life. Just as Confucius said: “At thirty, I had planted my feet firm upon the ground. At forty, I no longer suffered from perplexities.” Most of the middle-aged adults are leaders in every job and have increased family and job responsibilities. In China, middle age is the most stressful period of one's life because of job demands, caring for the older and younger generations, and paying for medical service and education. Studies on HRQOL among people with or without hypertension are limited in China. The aim of this study was to assess HRQOL as well as its sociodemographic and lifestyle determinants among middle-aged adults with or without hypertension.

## 2. Methods

### 2.1. Study Design

The study was conducted in Chongqing city in July 2009. This study used three-stage stratified random sampling method to recruit participants. Eligible participants were those born between 1956 and 1964 and were aged 45–53 years during time of the survey. At Stage 1, 10 districts and counties were randomly selected in Chongqing city. Stage 2 involved listing eligible villages within the selected districts and counties. In each selected district/count, about 8 to 10 villages were selected (Stage 2). About 10 to 15 participants were randomly selected in each village (Stage 3). In total, 1250 participated in the survey with a response rate of 98.4%. Of the 1250 participants, 6 were excluded in the analysis because of missing data.

### 2.2. Survey Administration

Face-to-face interview was conducted by senior medical school students. All of the participants were informed of the study's purpose, and their participation in the study was voluntary. The survey was conducted in compliance with the Ethical Committee of Chongqing Medical University. The questionnaire used in the study was divided into two sections. The first section collects information on sociodemographic and lifestyle factors and health conditions. Hypertension and other health conditions were assessed by the question “Have you ever been told by a doctor or other health professional that you have [disease or condition]?” Self-reported height and weight were collected to calculate body mass index (BMI). The second section was the short form (SF-36) survey. The questionnaire was piloted among 40 individuals in a hospital.

### 2.3. Outcome Measure: SF-36

A validated Chinese version of the SF-36 questionnaire is used in the study [14]. The questionnaire includes 36 questions; they were used to generate eight scales of HRQOL, namely, physical functioning (PF), role-physical (RP), bodily pain (BP), general health (GH), vitality (VT), social functioning (SF), role-emotional (RE), and mental health (MH). In the SF36 scoring system, the scales were assessed quantitatively, and a score is calculated based on the guidelines, with a higher score indicating a better state of health [15, 16].

### 2.4. Sociodemographic and Lifestyle Factors

Education was categorized into three groups: low (primary school), medium (junior school), and high (high school or above). Marital status was recorded as married and unmarried/divorced/widowed. Income was classified as low (<1600 Yuan/month) and high (>1600 Yuan/month). Job status was recorded as employed and unemployed/retired. Self-reported current living conditions were categorized as satisfactory and average/unsatisfactory. Smoking status and alcohol drinking were recorded into two categories (yes or no). Regular physical activity was categorized as usually and seldom/sometimes. Self-reported sleep quality was categorized as good and poor/average. Perception of family relationship and friend relationship was classified as harmony or poor/average. Childhood breastfeeding history was also assessed and categorized as yes or no.

### 2.5. Data Analysis

The data gathered from the questionnaires were carefully examined before inputting the data to the database using EpiData 3.1 software. The data were meticulously sorted, cleaned, and analyzed using Statistical Analysis System software (version 9.1, SAS Institute, Cary, NC). Chi-square test was used to compare differences between categorical variables, and* t*-test was conducted to compare differences in continuous variables between hypertensive and nonhypertensive groups. Multiple linear regression analyses were used to probe factors associated with domains of health-related quality of life among those with hypertension [17]. Sociodemographic and lifestyle factors included in the final multivariable regression models were based on findings from univariate analyses.

## 3. Results

Of the 1,224 participants, 150 (12.25%) reported having hypertension.  Table 1 showed the sample characteristics by hypertension status. Men were more likely to have hypertension than women. Significant differences were found with respect to marital status (*p* = 0.038), smoking (*p* = 0.0002), sleep quality (*p* = 0.049), BMI (*p* < 0.001), and history of breast feeding (*p* = 0.011) between hypertensive and nonhypertensive groups.

Participants with hypertension had a lower score of physical functioning, role-physical, bodily pain, general health, vitality, and social function than those without the condition (Table 2). No difference of role-emotional and mental health was seen between hypertensive and nonhypertensive groups.

In multivariable analyses, several sociodemographic and lifestyle factors were associated with domains of SF36 among those with hypertension (Table 3). Compared with women, men had a lower score of role-physical. Low education was associated with a lower score of physical functioning and bodily pain but not the others. Marital status was not significantly associated with the domains of HRQOL. Compared with those employed, those unemployed had higher score of general health and had a lower score of social function. Compared with those with higher incomes, those with low incomes had a lower score of bodily pain and vitality. Nonsmokers had a lower score of bodily pain than smokers. Those who had poor sleep had a lower score of physical functioning than those with good sleep. Compared with those with a good relationship with family, those who had a poor relationship with the family had a lower score of physical functioning, role-physical, social function, and role-emotional. Nonbreastfeeding had a lower score of bodily pain than breastfeeding. Those who had a higher body mass index had a higher score of role-physical and mental health.

## 4. Discussion

Our research shows that hypertension associated with physical functioning, role-physical, bodily pain, general health, vitality, and social function. In this study, middle-aged hypertensive adults reported significantly lower scores in most of the SF-36 dimensions, which were consistent with studies in other populations [18]. These findings further confirm that hypertension significantly affects health-related quality of life [19] among middle-aged hypertensive adults. Further longitudinal studies are required to test for the presence as well as the direction of associations between physical functioning, role-physical, bodily pain, general health, vitality, and social function and hypertension among hypertensive patients.

This study found that men had a poor state of role-physical. This study stressed the importance of gender on role-physical, among hypertensive patients. To understand the gender differences, biological factors may explain some of the differences but the main explanation is presumably gender disparities in work, economy, daily living, social life, and expectations between women and men [20]. More quantitative studies are needed to determine the association between gender and role-physical among hypertensive patients.

This study found that low education was associated with a lower score of physical functioning and bodily pain. Hypertensive patients with a low education level had a poorer state of lower score of physical functioning and bodily pain than those with a high education level. Educational attainment may influence the acquisition of knowledge about appropriate health practices, which may facilitate or constrain one's ability to maintain good physical function [21]. It may also due to the different social life and expectations. The link between education and HRQOL may be mediated by health literacy. Lifestyle intervention including weight reduction, physical activity, and healthy diet is beneficial for the management of hypertension [22, 23]. These healthy behaviors are often influenced by education.

This study found that among those with hypertension unemployment had a poorer social function and better state of general health than employment. This finding agrees with earlier research studies that unemployment and low socioeconomic status are associated with poor HRQOL [24, 25]. Previous study shows that job strain was significantly associated with vitality [26]. The results provide justification for further investigating the role of jobs as a risk factor for health-related quality of life among hypertensive patients.

Smoking has been associated with many adverse health outcomes including hypertension. Surprisingly, in our study we found that nonsmokers had a poor state of bodily pain than smokers, and we did not see a significant association between smoking and domains of SF36 except bodily pain among those with hypertension. Possible reasons are that quality of life rating is subjective and relative to a person's life expectations [27], influenced by a patient's strategies to cope with them [7]. Patients with hypertension who were smokers might feel contented and downscale their expectations for life and as long as they could stabilize their condition and be free from complications [28]. Further research is needed to assess whether quitting smoking may improve quality of life among those with hypertension in Chinese populations.

Previous studies have demonstrated that poor sleep was associated with poor health condition [28, 29]. We found that poor sleep quality was associated with poor state of physical functioning. Existing evidence suggests that there is a bidirectional association between obstructive sleep apnea and hypertension. Addressing sleep problems may improve quality of life among those with hypertension.

Our study stresses the importance of the relationship with family/colleagues/friends on health-related quality of life among hypertensive patients. This study found that those had a poor relationship with the family had a poorer state of physical functioning, role-physical, social function, and role-emotional than those with good relationship with family. It has been suggested that social support from family was strongly associated with hypertension treatment compliance [30] and better survival, lower depression, and higher compliance to medication [31]. Social support is the complex network of how a person gets and gives information and aid, as well as how they meet their emotional needs [32]. A good relationship with family and friends may enhance the social support and improve quality of life.

The beneficial effects of breastfeeding are well-known. Previous study showed that infant feeding patterns are associated with cardiovascular structures and function in childhood [33]. In this study, we found that hypertensive participants who were nonbreastfeeding had a poorer bodily pain among hypertensive patients than those who were breastfeeding. This study stressed the importance of breastfeeding on bodily pain among hypertensive patients.

This study found that body mass index was associated with role-physical and mental health among hypertensive patients. Hypertensive participants who have higher body mass index have a better state of role-physical and mental health. A previous study showed that the class I obese was significantly associated with better HRQL scores in the mental component summary than the normal weight in adults of the Chinese general population [34]. Some research presented that underweight was associated with poorer HRQL [35]. Previous studies showed the survival benefit or improved HRQL in the elderly or patients with existing chronic diseases who are overweight and moderately obese [36, 37]. This study stressed the importance of body mass index on role-physical and mental health among hypertensive patients.

This study has several limitations that should be noted. First, hypertension was based on self-reported doctor's diagnoses. According to the 2015 Chinese chronic disease and nutrition report, the prevalence of awareness was 44.2% among those aged 45–59 years [3]. In China, the prevalence of undiagnosed hypertension is high. Self-reported hypertension may not be accurate; many people with hypertension do not clearly know that they have hypertension in China. Hypertensive participants in this study may be relatively heavy, and this may lower the quality of life of hypertensive participants. And hypertensive patients with no obvious symptom may be mistaken for nonhypertensive patients. This may lower the quality of life of nonhypertensive participants. Second, we cannot establish any causation due to the cross-sectional design. Further longitudinal studies will be required to test for the presence of associations between demographics and the HRQOL and to fully interpret their clinical significance. Third, the study sample used in the investigation was relatively homogeneous regarding race/ethnicity. Future investigations with more heterogeneous samples are warranted. Fourth, the participants are middle-aged, and the findings may not apply to the younger or older individuals.

## 5. Conclusions

Hypertensive respondents with a high education, married, breastfeeding, and with higher incomes, good quality of sleep, positive relationship with family, and higher body mass index have better HRQOL in middle-aged people with hypertension. Respondents who currently do not smoke have poorer HRQOL in middle-aged people with hypertension. Those unemployed had a better state of general health and had a poorer state of social function. This study provides detailed information of the implications for health care providers to gain a more complete picture of their hypertension patients' health. What is more, to determine causal relationships, further longitudinal studies will be required to test for the presence of associations between sociodemographic and lifestyle and the HRQOL among hypertensive patients and to fully interpret their clinical and public health significance.

## Figures and Tables

**Table 1:** Characteristics of participants in Chongqing, China.

Characteristic	Hypertensive (*n* = 150)	Nonhypertensive (*n* = 1074)	*p* value
Gender (%)			<**0.0001**
Male	75.3	53.5	
Female	24.7	46.6	
Educational level (%)			0.499
Low	16.7	15.1	
Medium	45.3	50.5	
High	38.0	34.5	
Marital status (%)			**0.038**
Unmarried/divorced or separated/widowed	12.0	7.2	
Married or cohabitation	88.0	92.83	
Job conditions (%)			0.678
Unemployed/retired at home	52.7	54.5	
Employed	47.3	45.5	
Average monthly income (%)			0.232
<1600 Yuan	52.7	57.8	
>1601 Yuan	47.3	42.2	
Smoking (%)			**0.0002**
Yes	52.7	36.7	
No	47.3	63.3	
Alcohol drinking (%)			<**0.001**
Yes	28.7	45.5	
No	71.3	54.5	
Regular physical activity (%)			0.614
Seldom/sometimes	78.7	76.8	
Usually	21.3	23.2	
Have a regular daily life (%)			0.525
Seldom/sometimes	94.7	93.3	
Usually	5.3	6.7	
Sleep quality (%)			**0.049**
Good	32.7	41.1	
Poor/average	67.3	58.9	
Perception of family relationships (%)			0.597
Harmonious	74.0	76.0	
Poor/average	26.0	24.0	
Perception of relationships with colleague or friends (%)			0.274
Harmonious	78.0	73.8	
Poor/average	22.0	26.2	
Current living conditions (%)			0.868
Satisfactory	53.3	52.6	
Unsatisfactory/average	46.7	47.4	
Body mass index (mean, SD)	24.792 ± 4.2638	23.112 ± 8.6582	<**0.001**
Childhood breastfeeding history (%)			**0.011**
Breastfeeding	73.3	82.0	
Nonbreastfeeding	26.7	18.0	

**Table 2:** Descriptive statistics of each domain of the SF-36 among adults in Chongqing, China (mean, SD).

Domains	Hypertensive (*n* = 150)	Nonhypertensive (*n* = 1074)	*p* value
Physical functioning (PF)	80.2 (21.9)	88.2 (15.4)	<**0.001**
Role-physical (RP)	80.7 (19.5)	85.3 (17.7)	**0.004**
Bodily pain (BP)	76.7 (18.4)	81.7 (17.8)	**0.001**
General health (GH)	57.4 (15.3)	62.0 (14.5)	**0.000**
Vitality (VT)	61.1 (15.2)	65.9 (15.4)	**0.000**
Social function (SF)	80.3 (18.7)	84.8 (16.4)	**0.008**
Role-emotional (RE)	83.2 (14.7)	84.7 (17.0)	0.288
Mental health (MH)	66 (11.2)	67.0 (10.9)	0.310

Note: (1) values are mean and SD; (2) SD: standard deviation.

**Table 3:** Multiple linear regression analysis for the factors affecting health-related quality of life in hypertensive patients in Chongqing, China.

	PF		RP		BP		GH		VT		SF		RE		MH	
	*B* (s.e.)	*p*	*B* (s.e.)	*p*	*B* (s.e.)	*p*	*B* (s.e.)	*p*	*B* (s.e.)	*p*	*B* (s.e.)	*p*	*B* (s.e.)	*p*	*B* (s.e.)	*p*
Men versus women	−0.8 (5.4)	0.888	−10.8 (4.4)	**0.015**	−6.6 (4.5)	0.147	3.5 (4.1)	0.398	−4.4 (4.0)	0.277	−5.9 (4.34)	0.173	−1.9 (4.3)	0.664	0.8 (2.9)	0.779
Low education versus high education	−14.2 (6.2)	**0.023**	−6.5 (5.0)	0.194	−12.1 (5.4)	**0.028**	−4.8 (5.0)	0.345	1.3 (4.8)	0.781	−4.1 (5.3)	0.441	−4.5 (4.9)	0.353	−2.0 (3.5)	0.574
Medium education versus high education	2.2 (4.5)	0.621	0.4 (3.7)	0.922	−4.1 (4.0)	0.306	−2.3 (3.6)	0.529	5.9 (3.6)	0.100	5.7 (3.9)	0.145	0.0 (3.6)	0.993	2.0 (2.6)	0.457
Unmarried/divorced or separated/widowed versus married	−4.6 (5.5)	0.399	−4.8 (4.4)	0.283	−2.0 (4.8)	0.673	−3.9 (4.5)	0.387	−7.5 (4.2)	0.078	−8.7 (4.9)	0.079	−3.1 (4.3)	0.470	−1.9 (3.1)	0.539
Unemployed versus employed	−5.0 (3.7)	0.179	−7.3 (3.0)	0.017	−0.8 (3.2)	0.801	6.6 (2.9)	**0.025**	−0.1 (2.8)	0.960	−7.4 (3.1)	**0.018**	−3.9 (2.9)	0.191	−0.1 (2.1)	0.967
Low income versus high income	−5.0 (4.4)	0.254	−6.9 (3.5)	0.055	−7.8 (3.9)	**0.047**	−1.7 (3.5)	0.630	−9.4 (3.5)	**0.007**	−3.0 (3.8)	0.427	−5.5 (3.5)	0.119	−1.3 (2.5)	0.615
Nonsmokers versus smokers	−3.9 (4.3)	0.371	−3.4 (3.5)	0.338	−7.7 (3.8)	**0.044**	−0.5 (3.4)	0.883	−4.7 (3.3)	0.163	−3.3 (3.6)	0.361	1.0 (3.4)	0.758	0.8 (2.5)	0.760
Poor/average sleep quality versus good sleep quality	−8.8 (3.8)	**0.023**	−2.9 (3.0)	0.346	−1.6 (3.3)	0.634	−5.9 (3.0)	0.053	−3.0 (3.0)	0.306	−3.6 (3.2)	0.268	−5.8 (3.0)	0.056	−3.6 (2.2)	0.100
Poor/average versus good family relationship	−11.3 (4.7)	**0.018**	−7.8 (3.8)	**0.042**	−8.7 (4.2)	0.039	−7.3 (3.9)	0.061	−4.1 (3.7)	0.268	−14.5 (4.2)	**0.001**	−8.7 (3.7)	**0.021**	−4.3 (2.7)	0.117
Poor/average versus good relationship with colleague or friend	3.1 (5.4)	0.574	−2.6 (4.3)	0.543	4.0 (4.8)	0.408	3.7 (4.4)	0.406	0.6 (4.2)	0.880	7.8 (4.8)	0.110	1.0 (4.2)	0.822	1.4 (3.1)	0.652
Nonbreastfeeding versus breastfeeding	−7.1 (4.2)	0.097	−2.1 (3.4)	0.548	−7.7 (3.8)	**0.043**	0.6 (3.4)	0.859	−3.5 (3.3)	0.296	−5.9 (3.7)	0.114	−2.8 (3.4)	0.399	−0.9 (2.4)	0.706
Body mass index	0.5 (0.6)	0.364	1.1 (0.5)	**0.01**	0.4 (0.3)	0.264	0.2 (0.3)	0.47	0.5 (0.3)	0.147	1.1 (0.3)	0.00	0.8 (0.5)	0.107	0.6 (0.2)	**0.010**

Note: (1) PF: physical functioning; RH: role-physical; BP: bodily pain; GH: general health; VT: vitality; SF: social function; RE: role-emotional; MH: mental health; (2) potential independent variables for the multiple linear regression analysis include gender (1 = male; 2 = female (reference category)); educational level (1 = low; 2 = medium; 3 = high (reference category)); marital status (0 = unmarried/divorced or separated/widowed; 1 = married (reference category)); job conditions (0 = unemployed; 1 = employed (reference category)); average monthly income (0 = ⩽1600 Yuan; 1 = >1601 Yuan (reference category)); smoking (0 = nonsmoker; 1 = smoker (reference category)); sleep quality (0 = poor or average sleep quality; 1 = good sleep quality (reference category)); condition of getting along with family (0 = poor or average condition of getting along with family; 1 = harmony condition of getting along with family (reference category)); condition of getting along with colleague or friend (0 = poor or average condition of getting along with colleague or friend; 1 = harmony condition of getting along with colleague or friend (reference category)); feeding procedure (0 = nonbreastfeeding; 1 = breast feeding (reference category)).
